# Eosinophilic Dermatoses: Cause of Non-Infectious Erythema after Volume Replacement with Diced Acellular Dermal Matrix in Breast Cancer?

**DOI:** 10.3390/life14050608

**Published:** 2024-05-09

**Authors:** Jean Schneider, Seung Taek Lim, Yeong Yi An, Young Jin Suh

**Affiliations:** 1 School of Medicine, Texas Tech University Health Science Center, Lubbock, TX 79430, USA; jsschneider4055@gmail.com; 2 Division of Breast and Thyroid Surgical Oncology, Department of Surgery, The Catholic University of Korea St. Vincent’s Hospital, Suwon 16247, Republic of Korea; in-somnia@hanmail.net; 3 Department of Radiology, The Catholic University of Korea St. Vincent’s Hospital, Suwon 16247, Republic of Korea; didi97@catholic.ac.kr

**Keywords:** erythema, breast cancer, acellular dermal matrix, red breast syndrome, eosinophilic dermatoses

## Abstract

Introduction: Non-infectious erythema, or Red Breast Syndrome (RBS), has been observed on the skin where acellular dermal matrix was implanted, although the exact cause is yet to be determined. Patients and Methods: A total of 214 female patients underwent breast-conserving surgery (BCS) and volume replacement using diced acellular dermal matrix (dADM) for breast cancer between December 2017 and December 2018. After collecting and evaluating relevant clinical data, inflammation markers, along with NK cell status presented by IFN-γ secretion assay, were measured using ELISA. Results: Nineteen patients (8.88%) presented with RBS after BCS and dADM use. A significant increase of platelet-to-lymphocyte ratio was noted in the non-RBS group (*p* = 0.02). Compared to the RBS group (*p* = 0.042), the WBC level of the non-RBS group showed significant decrease over time. Eosinophil counts increased significantly at follow-up but went up higher in the RBS group. Multivariate analysis showed preoperative chemotherapy significantly increased the hazard of RBS (OR 3.274, *p* = 0.047 and OR 17.098, *p* < 0.001, respectively). Discussion: Though no causal relationship between RBS and immune status was proven, the results suggest an association between preoperative chemotherapy and RBS in addition to the possible role of eosinophilia in leading to eosinophilic dermatoses, which warrants further exploration and elucidation.

## 1. Introduction

The introduction of oncoplastic breast surgery (OPBS) has made it possible for patients to avoid cosmetic defects resulting from the removal of breast tissue as part of breast cancer treatment [1].

Previously, we reported initial cases of breast cancer patients who had undergone breast conserving surgery followed by inserting acellular dermal matrix into the cavity after removing index tumor [2]. Compared to using an implant or mastopexy with complex design, a more cost effective and convenient reconstruction (with better results that spare preoperative natural mammary ptosis with time and symmetry) is to use a diced acellular dermal matrix (dADM) of human origin which is implanted into the empty cavity immediately after the removal of the breast cancer [3,4,5,6]. There have been reports of non-infectious erythema described as “red breast syndrome” (RBS) in plastic surgery where implants are placed after a mastectomy [7,8,9,10,11,12,13], in which all breast parenchyma is removed, but there have been no studies on the incidence of RBS and the factors associated with it after breast-conserving surgery for breast cancer and placement of a new form of ADM, dADM, for volume replacement in the dead space where the cancer was removed. It cannot be claimed that dADM is the best material for volume replacement, but it may have significant advantages among the materials currently available. Therefore, it is very important to differentiate RBS from the infection, and it should be common practice to give adequate treatment aside from empirical antibiotics therapy, which is not helpful to alleviate non-infectious erythema.

The exact incidence of ‘Red Breast Syndrome (RBS)’ is unknown, but some estimations between 5~10% are reported [7,13], while the infection rate related to breast conserving surgery (BCS) with surrounding tissue or artificial material such as mesh is reported to be about 11% [14]. However, non-infectious erythema or RBS, which been noted to manifest unexpectedly after ADM use, involves local heat and redness on the skin over the dADM implantation site and is refractory to antibiotic therapy [7,8]. Various reports have suggested that this is due to endotoxins [9], the use of preservatives [7], neovascularization [10], delayed hypersensitivity reaction [8], or graft vs. host reaction [11]. But because dADM has been processed and irradiated in order to theoretically eradicate antigenicity, we suspect that the patient’s immunity may play a role when non-infectious erythema occurs in these RBS cases [12].

This study aims to identify the cause of this potentially bothersome problem by elucidating a possible relation between dADM and breast tissue in terms of immunological status at the time of diagnosis and follow-up.

## 2. Patients and Methods

The study protocol was reviewed and approved by the institutional review board of the Catholic University of Korea (VC17OESI0168, VC20RESI0225). This clinical trial is registered as KCT0003886 on the site of Clinical Research Information Service of Korea that is participating as one of the primary registries in the WHO International Clinical Trials Registry Platform. The study involved a total of 214 breast cancer patients treated between December 2017 and December 2018. All patients had resectable female breast cancer without suspicious skin invasion, regardless of the size or location of the index tumor. Exclusion criteria were inflammatory or infectious disease within a month before the surgery, autoimmune disease, and blood clotting disorders. Written informed consent was obtained for the use of dADM to fill the defect after removal of the breast cancer and the storage of clinical information in the database, its use for the purpose of this study only. All patients in the study underwent standard breast-conserving surgery. Volume replacement was performed by filling with dADMs inserted into the empty space left after the resection was completed, and the skin was sutured [3]. Diced acellular dermal matrix is a unique form of ADM was used to fill the dead space after tumor removal in the breast to reconstruct during breast-conserving surgery for cancer; the details of its production and sterilization process have been reported elsewhere [12]. All procedures were performed in accordance with the ethical standards of the institutional and/or national research committee and with the Helsinki Declaration of 1964 and its later amendments.

We collected electronic clinical data from patients that had dADM (Megaderm^®^, L&C Bio, Seongnam, Republic of Korea), inflammation markers such as neutrophil-to-lymphocyte ratio (N/L), platelet-to-neutrophil ratio (P/N) and platelet-to-lymphocyte ratio (P/L), and natural killer (NK) cell status using a quantitative sandwich ELISA (Enzyme-linked Immunosorbent Assay) kit to measure the released interferon-γ (IFN-γ) from natural killer (NK) cells to quantify NK cell activity. NK cell status was previously measured and collected using radioactive material such as ^51^Cr [15], but this approach is not used due to a longer time for the assay and greater funding requirements NK-IFN-γ secretion assay to determine NK cell status was performed by ELISA using NK Vue-Kit (NKMAX, Seongnam, Republic of Korea). Fresh whole blood (1 mL) was obtained using tubes containing Promoca (NKMAX, Seongnam, Republic of Korea). Promoca is a stimulatory cytokine that can specifically stimulate NK cells. The main cell population secreting IFN-γ after stimulating whole blood with Promoca was NK cells. After incubation at 37 °C for 20–24 h, the samples were centrifuged at 11,500× *g* for 1 min, and the supernatant was transferred to a 1.5 mL microtube, which was then stored at −20 °C until of IFN-γ levels reached the recommended amount according to the manufacturer’s instructions. Briefly, 50 µL of six standards, controls, and samples were incubated in an antihuman IFN γ-coated plate at room temperature for 2 h and washed with washing buffer. IFN-γ conjugate was added and further incubated at room temperature for 1 h. After washing and incubation with 100 µL of the substrate at room temperature for 30 min in the dark, the absorbance value was measured at 450 nm. Concentrations of IFN-γ were determined with a calibration curve. The measuring range was 40~2000 pg/mL and the total imprecision for two levels of controls was less than the 15% coefficient of variations. We differentiated wound infection from RBS, since RBS is defined as a type of erythema without identifiable pathogens. Based on previous reports of RBS, we used blood samples taken immediately after diagnosis as the baseline and compared results with blood samples taken at 6 months postoperatively, after all chemotherapy or radiation treatments that could affect postoperative blood tests had been completed. However, in cases where RBS occurred, blood was drawn to differentiate from infection, and this blood was used for further analysis to better understand the circumstances under which RBS occurred.

## 3. Statistical Evaluation

Categorical variables were reported as the number and percentages, and continuous variables were reported with mean ± standard deviation. The normality of distribution of continuous variables was tested by the Shapiro–Wilk or Kolmogorov–Smirnov test, and variance equality was assessed by Levene’s test. The comparison of continuous variables between groups was assessed using the student’s *t*-test or Mann–Whitney U-test. The chi square of Fisher’s exact test was used in categorical variables to assess the relationship between groups. Statistical analysis was performed using SPSS for Windows version 17.0 and a *p* value < 0.05 was accepted as statistically significant.

## 4. Results

This study is comprised of 214 female breast cancer patients treated between December 2017 and December 2018 (Table 1). Nineteen patients (8.88%) developed RBS within 6 months after breast-conserving surgery with dADM for reconstruction by the time of completion of systemic chemotherapy and/or external radiation treatment. Five out of 19 patients with RBS removed the dADM. Among 19 patients with RBS, 12 patients were premenopausal and seven were postmenopausal women. Two out of seven postmenopausal women with RBS had hypertension before surgery, while the others had no comorbid disease. In the RBS group, the index tumor was located in the upper outer quadrant in eight patients, in the upper inner quadrant in six, in the subareolar location in two, in the lower outer quadrant in two, and in the and lower inner quadrant in one case. Except for incision type and chemotherapy profile, there were no significant differences between the RBS and non-RBS groups, including age, menopausal status, BSA (body surface area), BMI (body mass index), TNM, breast volume, postoperative hormonal treatment status, tumor location, molecular subtype, and comorbid diseases [16].

Seven patients in the RBS group received neoadjuvant chemotherapy and had curative-intent operation. The number of patients who had adjuvant chemotherapy in the RBS group was 15. One patient did not receive systemic chemotherapy perioperatively. Of the nineteen patients, 17 patients developed RBS during systemic chemotherapy. The chemotherapeutic regimens were docetaxel–trastuzumab–pertuzumab (DHP) (one case), docetaxel–anthracycline–cyclophosphamide (TAC) (eight cases), and docetaxel–cyclophosphamide (TC) (nine cases). Two patients, who received three cycles of preoperative palliative chemotherapy with a triweekly TC regimen because of suspected bone metastasis, developed RBS on completion of three cycles postoperatively (approximately 8 weeks postoperatively); one patient, who was in clinically more than partial remission after triweekly neoadjuvant DHP regimen, developed RBS 6 weeks postoperatively on completion of radiation therapy, which was started immediately after surgery; and the other six patients, who received three cycles of triweekly neoadjuvant TC regimen, developed RBS 2–3 weeks after the initiation of radiation therapy following the completion of three cycles of postoperative triweekly adjuvant TC regimen. Among the eight patients who received the postoperative triweekly adjuvant TAC regimen, three developed RBS after three cycles, two after four cycles, one after five cycles, and the remaining two patients developed RBS two weeks after starting radiation therapy. One patient, who received an adjuvant triweekly TC regimen after surgery, developed RBS 3 weeks after completing four cycles and just before starting radiation therapy.

No other malignancies including hematologic abnormalities were reported thus far. There were no cases of RBS more than 6 months after breast-conserving surgery.

NK activity represented by IFN-γ was not statistically different at diagnosis or at follow-up (6 months after operation) between the RBS and non-RBS groups (Table 2). Within the RBS group, there was no significant increase or decrease of NK or inflammation markers (N/L, P/N, and P/L). On the contrary, the P/L ratio significantly increased at follow-up compared to the initial value in the non-RBS group (*p* = 0.02). Otherwise, there were no significant changes from initial to follow-up, including NK value change, in the non-RBS group.

By differential count of CBC (Table 3), we found that all the values at initial and follow-up failed to show significant differences between the RBS and non-RBS groups. However, the levels of hemoglobin, hematocrit, platelets, and white blood cells decreased significantly at follow-up compared to the initial levels in the non-RBS group (*p* < 0.001, *p* = 0.001, *p* < 0.001, *p* = 0.042, respectively). All the decreased values remained within the reference range, indicating no clinically significant change. In the RBS group, platelet values decreased significantly at follow-up, but still remained within the reference range (*p* = 0.023). By the fraction of white blood cells, all cells except eosinophils showed no significant changes from the initial value to the follow-up. Eosinophil counts at follow-up were increased in the RBS group (*p* = 0.04) and the non-RBS group showed significantly increased levels of eosinophils (*p* < 0.001) but the fold of increase compared to the initial value was much higher in the RBS group compared to the non-RBS group. This suggests there may a role of eosinophils in the development of RBS without definite infection, which is supported by the non-significant changes of segmented neutrophils from the initial to the follow-up in both groups.

Between the RBS and non-RBS groups, the incision type seemed meaningfully different on univariate analysis (*p* = 0.015) (Table 4).

**Figure 1:**
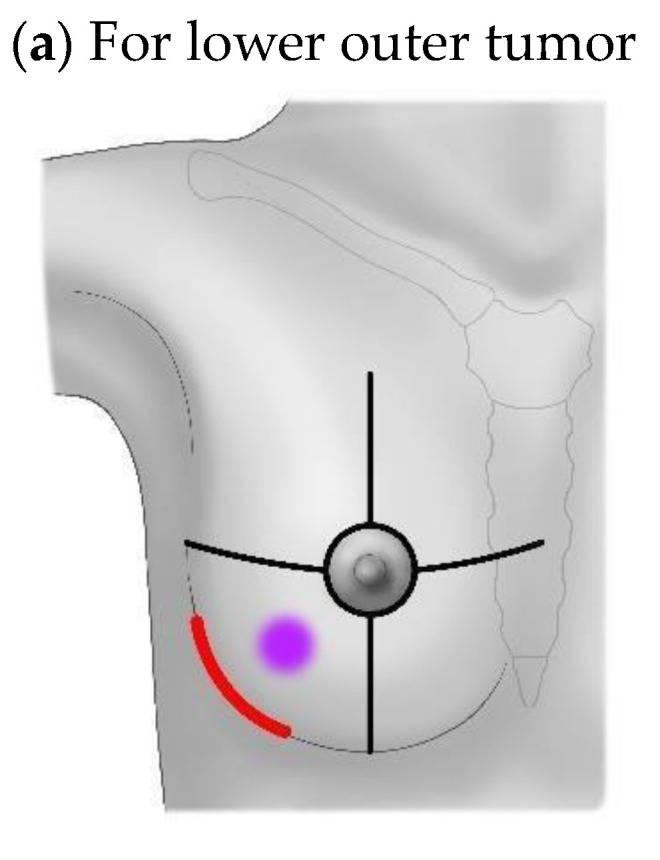

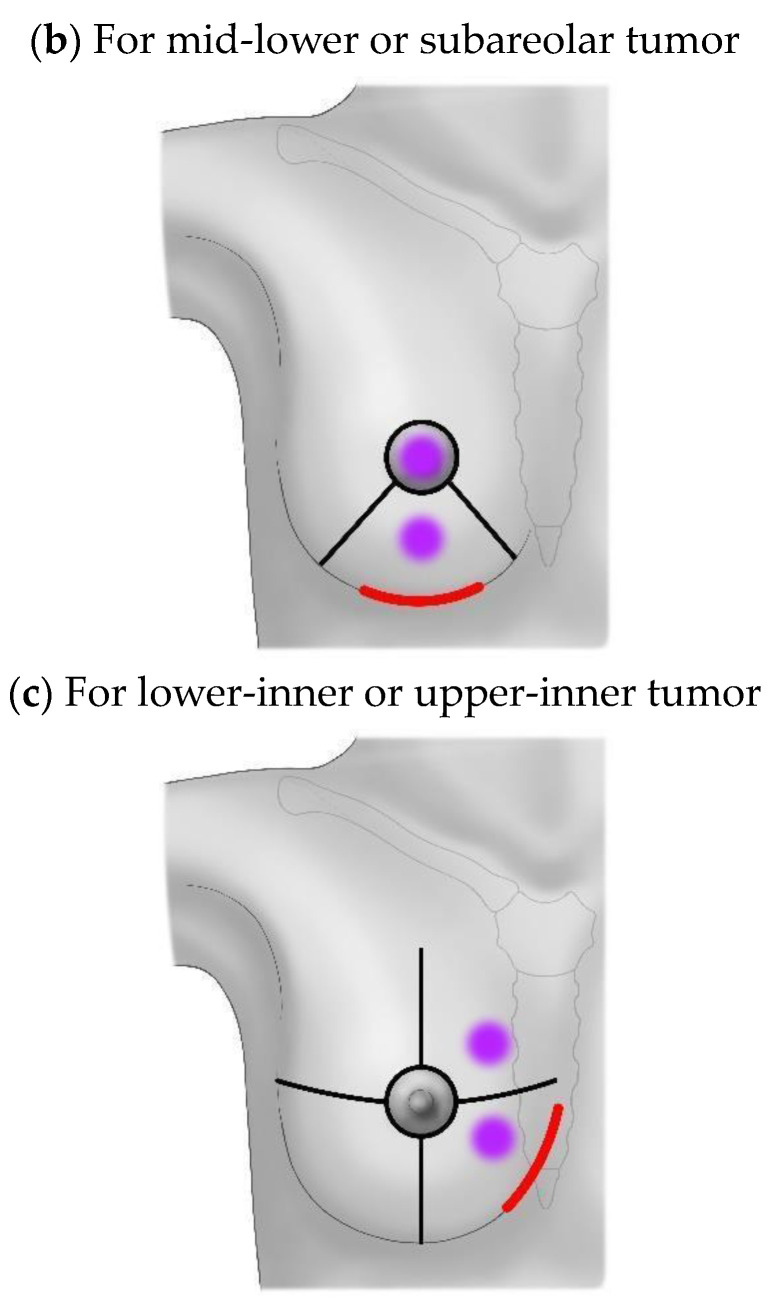
Peri-breast (Inframammary) incisions.

Radial incisions were more common in the RBS group, in contrast to all cases of peri-breast (inframammary) incisions that were utilized in the non-RBS group (Table 1) (Figure 1). Even though radial incision seems likely to pose much a higher odds ratio, leading to RBS on univariate analysis, there was no definite risk leading to RBS after radical incisions were examined on multivariate analysis (Table 5). Peri-breast incisions were made alongside the lowermost line of the breast including the inframammary line, according to the location of the index tumor, to reach through at the nearest point (Figure 1). Sentinel lymph node biopsy or axillary lymph node dissection was also carried out. Since the percentage of patients that received preoperative chemotherapy, either neoadjuvant or palliative, was relatively higher in the radial incision patients (two out of nine in the neoadjuvant group versus four out of nine in the palliative group), radial incision was a significant factor leading to RBS on univariate analysis. Even though patients with palliative chemotherapy seemed to be common in the RBS group, only one patient underwent preoperative palliative chemotherapy. After univariate and multivariate analyses, neoadjuvant and palliative chemotherapy preoperatively proved to be significant factors in provoking RBS, with an odds ratio of 3.274 (neoadjuvant, *p* = 0.047) and 17.098 (palliative, *p* < 0.001), respectively. Although these results have significance, the case numbers are not large enough to get an accurate statistical power. It should be evaluated in larger scale studies to get the definitive clinical meaning.

## 5. Discussion

Over the past few decades, there has been a significant shift in the surgical treatment of breast cancer from mastectomy to breast-conserving surgery, and this shift has been accelerated by the rapid introduction of the concept of oncoplastic surgery. In breast-conserving surgery, the cavity left after cancer removal is filled by volume replacement using adjacent breast parenchyma or adipofascial tissue, or if this is not possible, various materials have been used to fill the empty space at the index tumor site. In the latter case, there was no material that consistently showed a satisfactory outcome, but we devised a volume replacement method using diced acellular dermal matrix. Non-infectious erythema is not seen in cases where ADMs are not used and has been reported by many plastic surgeons who use ADM and have reported a number of possible mechanisms. However, no characteristic histologic findings have been described in these reports, and none of the proposed mechanisms have been proven to be causal.

We were unable to prove a direct relationship between NK activity at diagnosis and RBS after breast-conserving surgery and volume replacement with dADM for breast cancer [17,18,19]. Though there was no significant chronological change since diagnosis, NK activity reflected by IFN-γ secretion seemed to be decreased in the RBS group at follow-up, contrary to the increased IFN-γ secretion in the non-RBS group. This should not be interpreted definitively using only the data collected thus far. It may be due to the reflection of local tissue reaction during development of RBS, but there are likely to be other factors to be considered. Average values of the N/L, P/N, and P/L ratio at the time of diagnosis and follow-up, as well as chronological changes within each group, are shown in Table 2. These values are commonly used to indirectly evaluate patients’ immunological status or prognostic/predictive value in various solid tumors including breast cancer [20,21]. The initial and follow-up ratio of N/L, P/N, and P/L between the RBS and non-RBS groups was not statistically different, except that the platelet/lymphocyte ratio was significantly increased at follow-up compared to the initial ratio in the non-RBS group (*p* = 0.02). Considering the fact that many more patients with preoperative systemic chemotherapy were included in the RBS group, it may harbor some clinical meaning. But statistical evaluation was not possible due to the small number of such patients [22,23].

In this study, there were no significant differences in eosinophil counts between the RBS and non-RBS groups at the time of diagnosis or follow-up. However, the RBS and non-RBS groups showed significantly increased eosinophils at follow-up, compared to respective initial values. In the context of eosinophilia, the increment of eosinophil counts at follow-up was 2.99 times in the RBS group and 1.57 times in the non-RBS group, compared to initial values, respectively. Eosinophils act to defend against infectious stimuli, especially parasites, and play key roles in various immune-mediated skin and constitutional diseases such as allergic inflammation. During this defense mechanism, eosinophils release mediators that act in immune regulations as well as mediate skin symptoms [24]. Eosinophilic skin diseases show eosinophilic infiltration that may or may not accompany eosinophilia. Idiopathic eosinophilic dermatoses are known to be accompanied by eosinophilic infiltration that can affect certain tissue layers or adnexal structures of dermis, subcutaneous fat or other structures [25].

Initially, antibiotics were given to alleviate suspected infection; however, this had a minimal effect when there was no isolable pathogen from culture by aspiration under the erythema. Once we were unable to identify any specific pathogens causing the erythema, we began to remove dADM to minimize further subcutaneous fat necrosis that might lead to devastating skin necrosis. Most of the RBS cases that required surgical management showed profuse subcutaneous fat necrosis at re-exploration. Once the dADM was removed, the skin erythema began to disappear within a couple of weeks in most cases. Even though it is difficult to prove that there is a direct causal relationship with all these findings, we suspect that dADM may activate the immune system and affect specific skin layers, such as dermis or subcutaneous fat, to induce allergic reaction and cause eosinophilic dermatitis. Therefore, we suspect that the removal of dADM can lead to fast recovery from RBS leaving no sequelae behind, because it should be the triggering factor of the non-infectious erythema. Taken together with the increment of eosinophil counts in the non-RBS group, dADM may induce the host immune system by recruiting eosinophils over the area where dADMs are stacked. Nevertheless, most patients undergoing breast-conserving surgery and volume replacement with dADM can recover from this rare eosinophilic dermatitis without the need of removing the dADM [26]. Preoperative chemotherapy and granulocyte stimulating factors used to facilitate recovery from neutropenia after chemotherapy may affect the host immune system, especially during the course of systemic treatment [27,28]. The analysis of many more cases of RBS should be required to determine an objective causal relationship between eosinophils and RBS after breast-conserving surgery and volume replacement with dADM. Lastly, no case of any other malignancies, including hematologic diseases such as anaplastic large cell lymphoma, has been seen after certain synthetic breast implant insertion for reconstruction, for more than 3 years since the first case of dADM reconstruction with breast-conserving surgery for breast cancer [29,30].

## 6. Conclusions

We believe that, in cases of suspected red breast syndrome, it is important to first differentiate whether there is an infection or not; if RBS is confirmed, unnecessary antibiotics should be avoided, and short-term steroid use can effectively relieve symptoms. In this study, we proposed eosinophilia as a possible reason for the development of RBS after volume displacement with dADM after conventional breast-conserving surgery. To our knowledge, this is the first report of de novo RBS in patients undergoing partial mastectomy for resectable breast cancer and the implantation of a dADM as volume displacement.

## Figures and Tables

**Table 1:** Patients’ characteristics.

			RBS (*n* = 19)	Non-RBS (*n* = 195)	*p* Value
Age			47.9474 ± 9.38364	51.8718 ± 8.83053	0.067
Menopausal status	Premenopausal		12 (63.2%)	121 (62.1%)	0.924
	Postmenopausal		7 (36.8%)	74 (37.9%)	
Tumor location	UOQ		8 (42.1%)	72 (36.9%)	0.994
	UIQ		6 (31.6%)	63 (32.3%)	
	LOQ		2 (10.5%)	21 (10.8%)	
	LIQ		1 (5.3%)	16 (8.2%)	
	SA		2 (10.5%)	23 (11.8%)	
Incision	Circumareolar		2 (10.5%)	28 (14.4%)	0.016
	Periareolar		13 (68.4%)	153 (78.5%)	
	Peri-breast		0 (0%)	9 (4.6%)	
	Radial		4 (21.1%)	5 (2.6%)	
Breast volume (cc)			959.1053 ± 355.751	1052.9795 ± 411.67598	0.339
Body surface area (m^2^)			1.6253 ± 0.12677	1.6405 ± 0.32539	0.840
Body mass index (Kg/m^2^)			25.5968 ± 3.40849	24.1507 ± 4.95705	0.216
Comorbid disease	DM	No	19 (100%)	183 (93.8%)	0.606
		Yes	0 (0%)	12 (6.2%)	
	Hypertension	No	17 (89.5%)	173 (88.7%)	0.921
		Yes	2 (10.5%)	22 (11.3%)	
	Other	No	19 (100%)	192 (98.5%)	0.756
		Yes	0 (0%)	3 (1.5%)	
Molecular subtype	LUM A		9 (47.4%)	130 (66.7%)	0.181
	LUM B		4 (21.1%)	24 (12.3%)	
	HER+		4 (21.1%)	19 (9.7%)	
	Triple negative		2 (10.5%)	22 (11.3%)	
TNM	0		2 (10.5%)	32 (16.4%)	0.109
	I		5 (26.3%)	84 (43.1%)	
	II		7 (36.8%)	62 (31.8%)	
	III		4 (21.1%)	13 (6.7%)	
	IV		1 (5.3%)	4 (2.1%)	
Chemotherapy	Neoadjuvant	No	12 (63.2%)	171 (87.7%)	0.01
		Yes	7 (36.8%)	24 (12.3%)	
	Adjuvant	No	4 (21.1%)	67 (34.4%)	0.240
		Yes	15 (78.9%)	128 (65.6%)	
	Neoadjuvant→Adjuvant	No	13 (68.4%)	172 (88.2%)	0.028
		Yes	6 (31.6%)	23 (11.8%)	
	Palliative	No	17 (89.5%)	188 (96.4%)	<0.001
		Yes	2 (10.5%)	7 (3.6%)	
Hormonal therapy	No		6 (31.6%)	40 (20.5%)	0.254
	Yes		13 (68.4%)	155 (79.5%)	

**Table 2:** NK cell activity and inflammation markers.

		**Initial**			**Follow** **Up**	
	**RBS (*n* = 19)**	**Non-RBS (*n* = 195)**	***p* Value**	**RBS (*n* = 19)**	**Non-RBS (*n* = 195)**	***p* Value**
NK	1168.326316 ± 728.3503391	913.436096 ± 718.6330553	0.143	868.878947 ± 691.2452641	949.281482 ± 728.2787949	0.646
N/L	1.737826 ± 0.8557119	1.952532 ± 0.9806427	0.358	1.937236 ± 1.0851416	2.157756 ± 1.2841531	0.470
P/N	108.801468 ± 76.5852187	105.300069 ± 160.8408602	0.925	98.267985 ± 42.4433964	87.012974 ± 35.0998656	0.192
P/L	157.625805 ± 81.4910094	152.905757 ± 56.0619944	0.738	159.601787 ± 58.3481064	168.268940 ± 72.2833186	0.613
	**RBS (*n* = 19)**			**Non-RBS** **(*n* = 195)**		
	**Initial**	**Follow** **Up**	***p* Value**	**Initial**	**Follow** **Up**	***p* Value**
NK	1168.326316 ± 728.3503391	868.878947 ± 691.2452641	0.202	913.436096 ± 718.6330553	953.691935 ± 727.7360589	0.591
N/L	1.737826 ± 0.8557119	1.937236 ± 1.0851416	0.533	1.952532 ± 0.9806427	2.157756 ± 1.2841531	0.077
P/N	108.801468 ± 76.5852187	98.267985 ± 42.4433964	0.603	105.300069 ± 160.8408602	87.012974 ± 35.0998656	0.122
P/L	157.625805 ± 81.4910094	159.601787 ± 58.3481064	0.932	152.905757 ± 56.0619944	168.268940 ± 72.2833186	0.02

NK = natural killer cell activity. N/L = neutrophil to lymphocyte ratio. P/N = platelet to neutrophil ratio. P/L = platelet to lymphocyte ratio.

**Table 3:** Differential count of CBC.

		**Initial**			**Follow** **Up**	
	**RBS (*n* = 19)**	**Non-RBS (*n* = 195)**	***p* Value**	**RBS (*n* = 19)**	**Non-RBS (*n* = 195)**	***p* Value**
Hemoglobin (g/dL)	13.005263 ± 1.3554309	12.989231 ± 1.2428504	0.958	12.3842 ± 1.27160	12.4728 ± 1.38955	0.790
Hematocrit (%)	39.163158 ± 3.7985685	39.016769 ± 3.2851918	0.855	38.1053 ± 3.25891	37.8800 ± 3.17772	0.769
Platelet (×10^3^/µL)	273.947368 ± 61.0341195	274.943590 ± 73.1470902	0.954	227.6316 ± 59.00114	231.5128 ± 61.79700	0.793
White blood cell (×10^3^/µL)	5.613684 ± 1.5691548	6.033436 ± 1.6691522	0.294	4.8674 ± 1.33476	5.3612 ± 4.27633	0.618
Segmented neutrophil (×10/µL)	54.731579 ± 11.6569032	56.653846 ± 11.3055193	0.481	54.0211 ± 12.71345	57.8374 ± 9.62992	0.217
Lymphocyte (×10/µL)	35.694737 ± 10.0949643	33.380513 ± 9.8606492	0.331	32.8789 ± 10.05566	33.0754 ± 27.85745	0.976
Monocyte (×10/µL)	7.399474 ± 5.0950635	7.170256 ± 5.3150959	0.857	8.4053 ± 3.14245	8.0774 ± 4.67696	0.765
Eosinophil (×10/µL)	1.431579 ± 0.9189684	2.148205 ± 2.2124144	0.164	4.2895 ± 5.57752	3.3749 ± 3.71695	0.331
Basophil (×10/µL)	0.6089 ± 0.77994	0.4995 ± 0.44203	0.554	0.4053 ± 0.25050	0.4436 ± 0.38462	0.671
	**RBS (*n* = 19)**			**Non-RBS** **(*n* = 195)**		
	**Initial**	**Follow** **Up**	***p* Value**	**Initial**	**Follow** **Up**	***p* Value**
Hemoglobin (g/dL)	13.005263 ± 1.3554309	12.384211 ± 1.2715971	0.154	12.989231 ± 1.2428504	12.472821 ± 1.3895522	<0.001
Hematocrit (%)	39.163158 ± 3.7985685	38.105263 ± 3.2589149	0.353	39.016769 ± 3.2851918	37.880000 ± 3.1777188	0.001
Platelet (×10^3^/µL)	273.947368 ± 61.0341195	227.631579 ± 59.0011398	0.023	274.943590 ± 73.1470902	231.512821 ± 61.7970038	<0.001
White blood Cell (×10^3^/µL)	5.613684 ± 1.5691548	4.867368 ± 1.3347611	0.123	6.033436 ± 1.6691522	5.361174 ± 4.2763289	0.042
Segmented Neutrophil (×10/µL)	54.731579 ± 11.6569032	54.021053 ± 12.7134478	0.859	56.653846 ± 11.3055193	57.837436 ± 9.6299246	0.266
Lymphocyte (×10/µL)	35.694737 ± 10.0949643	32.878947 ± 10.0556551	0.395	33.380513 ± 9.8606492	33.075385 ± 27.8574466	0.885
Monocyte (×10/µL)	7.399474 ± 5.0950635	8.405263 ± 3.1424466	0.469	7.170256 ± 5.3150959	8.077436 ± 4.6769564	0.074
Eosinophil (×10/µL)	1.431579 ± 0.9189684	4.289474 ± 5.5775238	0.04	2.148205 ± 2.2124144	3.374923 ± 3.7169474	<0.001
Basophil (×10/µL)	0.608947 ± 0.7799351	0.405263 ± 0.2504966	0.290	0.499486 ± 0.4420275	0.443590 ± 0.3846238	0.184

**Table 4:** Univariate analysis.

		OR	95% CI	*p*-Value
Age		1.051	0.996–1.108	0.069
Menopause	Premenopausal	1		
	Postmenopausal	0.954	0.359–2.531	0.924
Tumor location	OUQ	1		
	UIQ	0.857	0.282–2.604	0.786
	LOQ	0.857	0.169–4.348	0.852
	LIQ	0.563	0.066–4.821	0.6
	SA	0.783	0.155–3.951	0.767
Incision type	Circumareolar	1		
	Peri-areolar	1.190	0.254–5.561	0.825
	Inframammary			
	Peri-breast	0	0	0.999
	Radial	11.200	1.600–78.400	0.015
Breast volume		0.999	0.998–1.001	0.338
Body surface area (m^2^)		0.830	0.135–5.102	0.84
Body mass index (Kg/m^2^)		1.071	0.964–1.190	0.203
Diabetes	No	1		
	Yes	0	0	0.999
Hypertension	No	1		HBP
	Yes	1.081	0.234–4.996	0.921
Other	No	1		
	Yes	0	0	0.999
Molecular subtype	Luminal A	1		
	Luminal B	2.293	0.655–8.031	0.194
	HER2	3.018	0.845–10.771	0.089
	Triple negative	1.303	0.264–6.438	0.745
TNM	0	1		
	I	0.952	0.176–5.159	0.955
	II	1.806	0.355–9.205	0.477
	III	4.923	0.801–30.253	0.085
	IV	4.000	0.292–54.715	0.299
Neoadjuvant CTx.	No	1		
	Yes	4.156	1.491–11.588	0.006
Adjuvant CTx.	No	1		Adjuvant CTx.
	Yes	1.963	0.627–6.149	0.247
Neo + Adjuvant CTx.	No	1		
	Yes	3.452	1.195–9.969	0.022
Palliative CTx.	No	1		
	Yes	19.532	5.986–63.733	<0.001
Hormonal Tx.	No	1		
	Yes	0.559	0.200–1.563	0.268

CTx = chemotherapy. Tx = therapy.

**Table 5:** Multivariate analysis.

		OR	95% CI	*p* Value
Incision type	Circumareolar	1		
	Periareolar	1.330	0.238–7.430	0.745
	Peri-breast	0	0	0.999
	Radial	5.125	0.497–52.887	0.17
Neoadjuvant chemotherapy	No	1		
	Yes	3.274	1.018–10.526	0.047
Palliative chemotherapy	No	1		
	Yes	17.098	5.060–57.767	<0.001

## Data Availability

Data supporting reported results can be found on electronic medical record database only, any kind of act related to share, gain, create related to that database is completely prohibited due to privacy or ethical restrictions according to the law and regulations of the Republic of Korea and institutional review board of the Catholic University of Korea St. Vincent’s hospital.
